# Thermal insensitivity of an ENZ-ITO clad, hollow-core micro-ring resonator

**DOI:** 10.1038/s41598-024-82147-7

**Published:** 2025-03-29

**Authors:** Andrew S. DeLoach, Stephen R. Anderson, Sang-Yeon Cho, Jimmy H. Ni, Weimin Zhou

**Affiliations:** DEVCOM Army Research Laboratory, 2800 Powder Mill Rd, Adelphi, MD 20783 USA

**Keywords:** Metamaterials, Microresonators

## Abstract

We report on the experimental demonstration of temperature insensitivity in an epsilon-near-zero (ENZ) indium-tin-oxide (ITO) cladded, hollow-core micro-ring resonator. The permittivity of the ITO cladding was engineered to a near zero value at 1550 nm wavelength to support index guiding in the air-ITO waveguide around the C band. The air-ITO ring-resonator structure was designed using numerical simulations to support whispering gallery modes. The hollow-core microresonator structure was fabricated using two-photon lithography to 3D print a sacrificial scaffold. Guided resonator modes were observed by coupling tunable laser light to the ring resonator using a tapered fiber. The demonstrated hollow-core microresonator exhibits athermal behavior and has a temperature dependent wavelength shift of 1 pm/°C.

## Introduction

Most state-of-the-art precision timing systems utilize a laser stabilized by optical transitions in an atomic source to achieve long-term stability with a precision oscillator^1,2^. One limitation of these systems is that the oscillator requires a very high quality factor (Q) cavity which is inherently susceptible to environmental perturbations such as temperature variations and vibrations. This can cause the generated frequency to drift with time unless the oscillator is isolated from environmental interference with extensive housing, significantly increasing the size, weight, power consumption, and cost (SWaP-C) of the system. The high SWAP-C of these devices restricts their widespread adoption, particularly in smaller portable device applications.

Nonlinear high-Q microresonator based optical frequency combs (OFC) have been demonstrated to generate low phase noise radio-frequency (RF) signals^3,4^. Currently, significant effort is being directed towards the combination of photonic integrated circuits (PICs) with an atomic source to produce chip-scale OFC based atomic clocks^5^. These are complex devices that perform the tasks of laser cooling, trapping, probing the atoms, and detecting the atomic transitions. Although this work is promising, there is an inherent increase in complexity when including atomic sources on chip.

We are pursuing an alternative method to achieving long term stability, that does not use an atomic source, but rather a chip-scale, temperature insensitive optical resonator cavity. This resonator could be used as the temperature insensitive frequency reference to lock a higher-Q microresonator in a chip-scale oscillator or clock system. This locking technique is similar to that of some commonly used phaselock loop systems that provide long term oscillator stability^6–8^. This would eliminate the need for high SWaP-C environmental isolation housing and the atomic source, as well as be CMOS process compatible, further lowering cost.

Athermal micro-ring resonators (MRRs) can be realized by adding temperature controlling feedback components^9^ onto the chip but this increases size and power consumption. Another way is to use materials with a very low thermo-optic coefficient (TOC), which describes the temperature dependence of the refractive index. One approach to obtain a low TOC is to combine positive and negative TOC materials to make athermal layered waveguides and resonators such as TiO_2_-Si_3_N_4_^10,11^, Si-Polymer^12,13^, or TiO_2_-LN^14^. One downside to this approach is that the devices are subject to tight fabrication tolerances and the TOC will be near zero for only a small wavelength range.

Our approach is to use an indium-tin-oxide (ITO) cladding over an air core because air has a negligible TOC compared to standard photonic waveguiding media like Si or Si_3_N_4_^15,16^. ITO is used as the cladding layer because ITO films have been shown to achieve a tunable epsilon-near-zero (ENZ) property using certain post deposition annealing procedures^17–20^. Devices can be engineered to operate within a large wavelength range due to the easy tunability of the ENZ regime of ITO. Additionally, the athermal property of an index guided, air core design will extend over a larger wavelength range than the layered hybrid structures since light is predominantly confined in the low TOC core anywhere the cladding index is lower that the core index.

Athermal properties due to the confinement of light primarily in an air core have been demonstrated in other hollow-core optical devices such as hollow-core fibers (HCFs)^21–23^. The guiding mechanism in HCFs is typically based on photonic bandgaps^24,25^ or antiresonance^26,27^, though simulations have shown that HCFs can also be made using an ENZ cladding material to index guide light^28^ and that ENZ materials can strongly confine light in the core^29^. Therefore, reduced thermal sensitivity of the resonant wavelengths in an ENZ clad, air-core MRR is expected since most of the resonant optical mode will be in the low TOC core and not in the cladding.

In this paper, we report the design and fabrication of an ENZ-ITO clad, hollow-core resonator and demonstrate the athermal behavior of the device within and around the C-band wavelengths. This device exhibits its athermal behavior due to light confinement in the hollow-core by the ENZ ITO cladding layer. Since ITO is a common material used in semiconductor processing, this work paves the way for developing an intrinsically temperature insensitive cavity that can be integrated with PICs. This device also demonstrates the potential of 3D printed optical devices and this method could be applied to other complex structures to allow for different mode confinement and dispersion control.

## Results

### Hollow core resonators

**Fig. 1:**
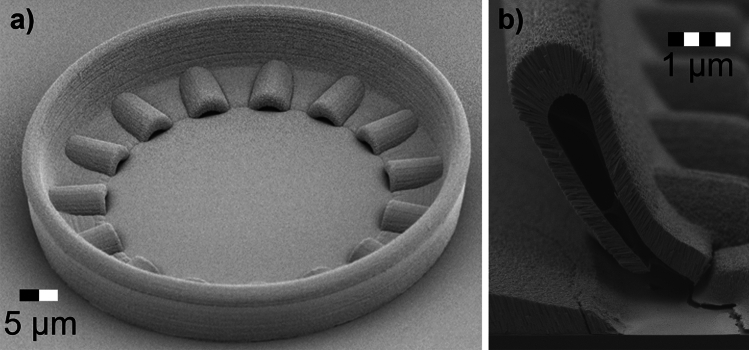
Device characterization. (**a**), SEM image of a resonator after IP-Dip removal. (**b**)**,** SEM image of the cross-section of a straight line structure showing the IP-Dip removal.

The core of the device was defined by a sacrificial scaffold fabricated out of Nanoscribe IP-Dip photoresin using a Nanoscribe Photonic Professional GT2 two-photon polymerization 3D printer. The ITO cladding was deposited onto this scaffold prior to the scaffold removal (Methods section). The scaffold was designed with a radius of 65 µm, a height of 14 µm, and a core thickness of 3 µm. Scanning electron microscopy (SEM) images of this device can be seen in (Fig. 1a). Another structure that shows the cross-section is shown in (Fig. 1b). This structure has the same photoresin drain features and cross-section as the resonator, but is a straight line which allows it to be cleaved and confirm that the IP-Dip was successfully removed from the core area. The geometry of the core in this line structure is expected to vary from the actual resonator due to the defects added during the cleave and annealing steps.

**Fig. 2:**
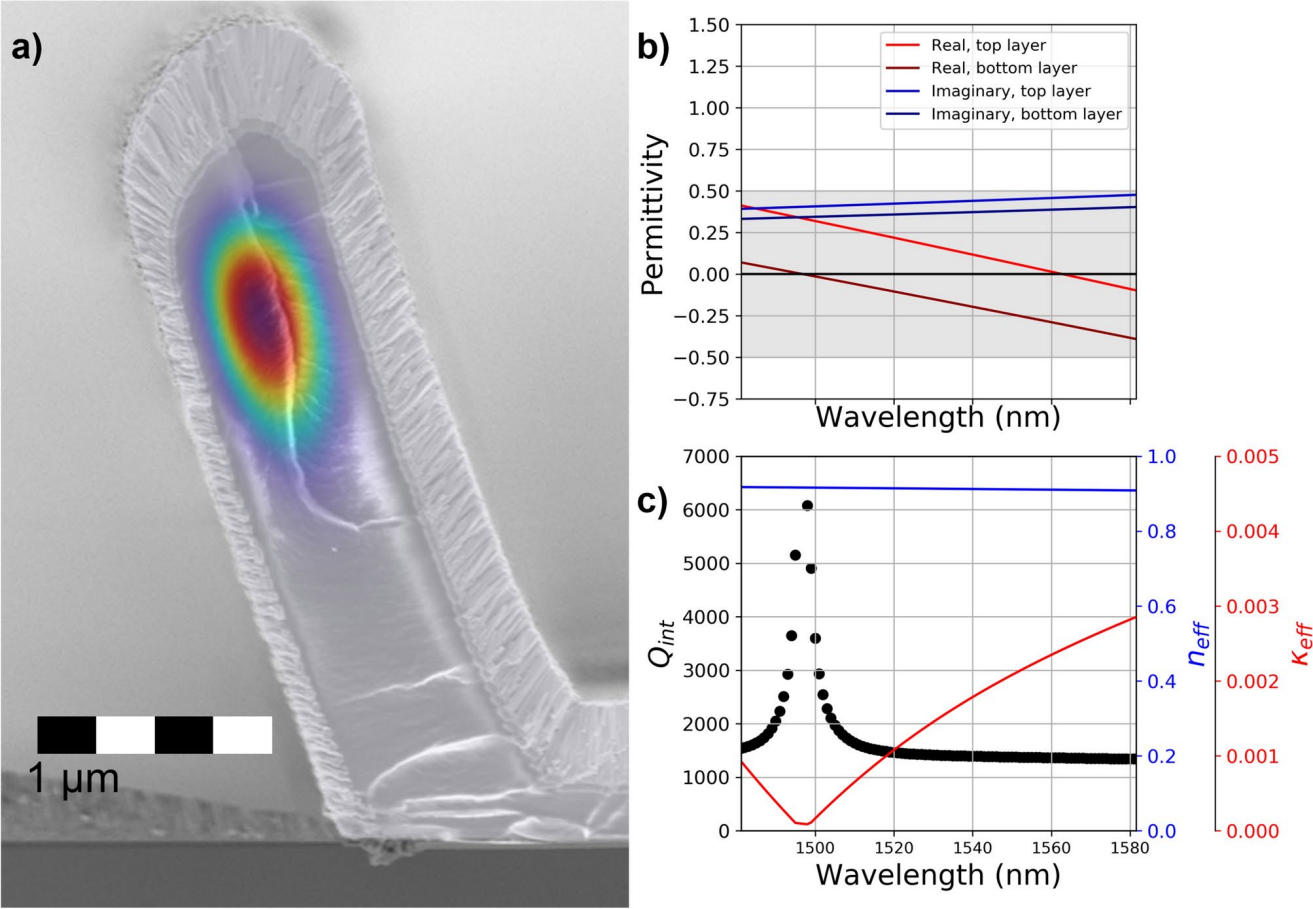
Guided mode calculations. (**a**), SEM image of the resonator cross-section. The IP-Dip was left in to prevent damage to the cross-section geometry during cleaving. Overlaid on the cross-section is the guided mode found from COMSOL simulation. (**b**), The spectroscopic ellipsometry of the ITO film is shown with a grey band indicating that ε_1_ and ε_2_ are in the ENZ regime over the entire tested wavelength range. The lighter color red and blue indicate the top layer, air exposed side of the film and the darker colors indicate the bottom layer substrate side. (**c**), The intrinsic Q (black line) with the real (blue line) and imaginary (red line) parts of the effective index as a function of wavelength.

In Fig. 2a, another cross-section is shown with an overlay of the norm of the field components, ${E}_{norm}=\sqrt{{E}_{x}^{2}+{E}_{y}^{2}}$, for the guided mode computed using COMSOL Multiphysics™. The IP-Dip was not removed in this cross-section so that the geometry of the core region could be preserved for the calculations. The COMSOL simulation used the real and imaginary permittivity data (ε_1_ and ε_2_, respectively) that were measured using spectroscopic ellipsometry (SE) taken from the processed ITO films as seen in (Fig. 2b). It is not possible to measure the exact ITO areas that make up the sides of the resonator, so this data was taken from ITO grown directly on the substrate near the resonators. We modeled the ITO film using a graded layer approach which models the full film as the sum of many thin slices. This method can describe variations in the optical parameters as a function of film depth which is needed when modelling thick ITO films^19,20^. The results indicate that ε_1_ and ε_2_ are entirely within the ENZ regime throughout the bulk of the film at the wavelengths tested (grey area in Fig. 2b).

From these calculations, the real and imaginary part of one fundamental guided mode’s effective index, ${\widetilde{n}}_{eff}={n}_{eff}+i{\kappa }_{eff}$, was derived as shown in Fig. 2(c). The real part is ~ 0.91 at 1550 nm wavelength, where the resonator has a free-spectral range (FSR) of ~ 6.4 nm. The intrinsic Q of this device as a function of wavelength was calculated from ${\widetilde{n}}_{eff}$ and these values are shown in Fig. 2c. This was done using the following Equation^30,31^:

1 $$Q=\frac{2\pi d}{\lambda \mathcal{L}}$$

where $\lambda$ is the wavelength, $d = 2\pi \left( {radius} \right) \cdot n_{{eff}}$ is the round trip distance, and $\mathcal{L}$ is the round trip fractional loss:

2 $$\mathcal{L}=1-exp\left(\frac{-4\pi {\kappa }_{eff}d}{\lambda }\right)$$

The intrinsic Q peaked above 6000 at 1498 nm which corresponds to where ε_1_ of the inner layers of the ITO cladding crosses zero. At this wavelength, the waveguide has the highest index contrast between the core and the cladding, therefore, the guided mode confinement in the air core is maximized. At lower wavelengths the index contrast is lower so more of the mode leaks into the cladding and loss increases. At higher wavelengths the ITO begins to act more metal like which causes more absorption loss. This analysis shows that with proper tuning of the ENZ property a device could potentially achieve a Q of 6000 or more.

It is likely that the values of ε_1_ and ε_2_ of the ITO cladding of the fabricated resonator differ from those of the ITO on the substrate where the ε_1_ and ε_2_ in Fig. 2b were measured. This is because the annealing parameters to achieve the proper ENZ condition are dependent on film thickness and interface materials which varies around the sides of the resonator and the fact that the ITO cladding is elevated which would change the annealing rate.

**Fig. 3:**
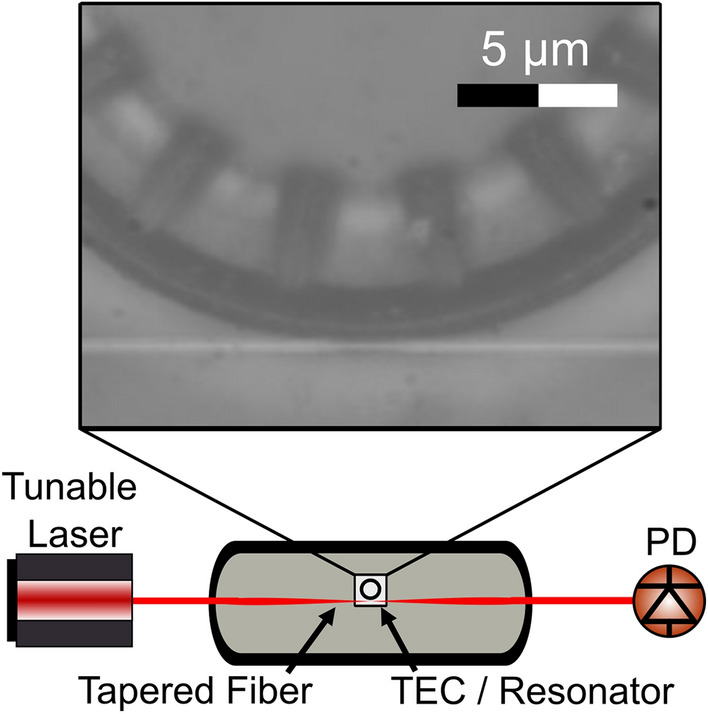
Experimental testing setup. Diagram of the experimental testing setup. The microring resonator was mounted onto a manipulator arm and then brought into range for coupling with the tapered fiber. A magnified image of this can be seen in the inset.

The experimental setup can be seen in (Fig. 3). The sample was mounted onto a thermo-electric cooler (TEC) which was then mounted onto a manipulator arm. Light was coupled into the device using a tapered fiber. This setup is described in more detail in the Methods.

### Coupling light into the resonator

**Fig. 4:**
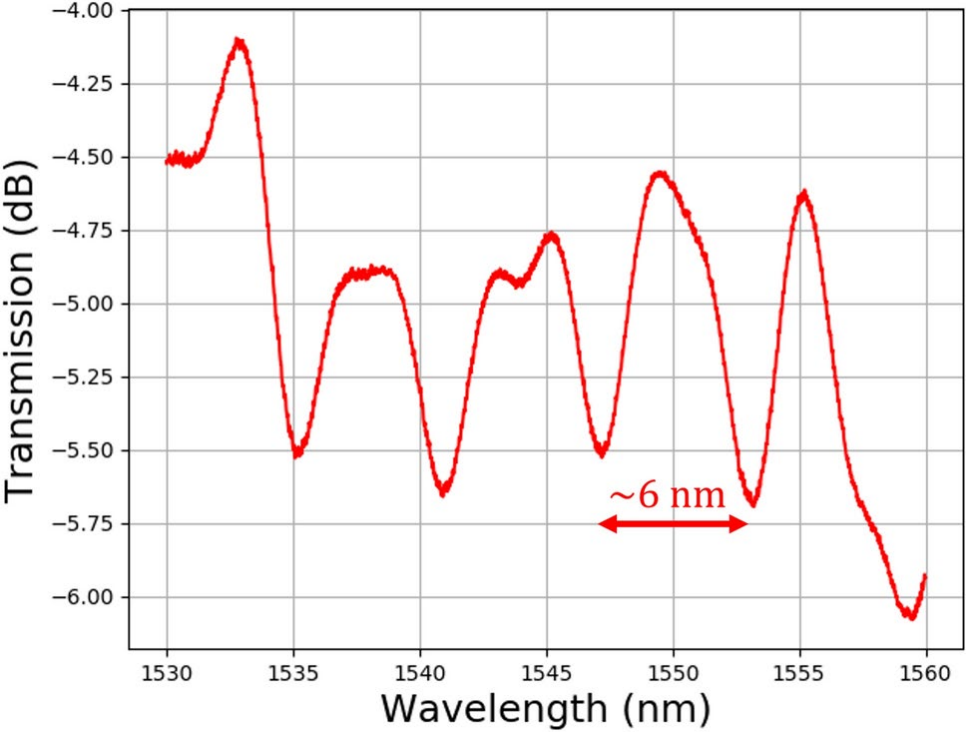
Experimental results of coupling light into the resonator. The transmission spectrum obtained by evanescently coupling the resonator to the tapered fiber. A baseline measurement was performed before the coupled measurement and subtracted from the coupled spectrum.

Figure 4 shows the transmission spectra of the tapered fiber coupling light into the resonator at 20 °C. The baseline uncoupled spectrum is subtracted from the dataset to remove the contribution of losses internal to the characterization equipment. The resulting spectrum shows a FSR of ~ 6.0 nm, which agrees with the calculated value for a resonator with similar geometry (radius of 65 µm) and effective index of approximately 0.91. The resonant dips vary from a single Lorentzian shape most likely due to transverse electric (TE) and transverse magnetic (TM) mode mixing and show a power loss of ~ 1 dB. At the resonance located at 1553 nm, the loaded Q value was extracted to be approximately 700. In previous studies of air-clad, solid-core resonators^32,33^, the tapered fiber can be in direct contact with the core material. Here, the tapered fiber can only contact the ENZ ITO cladding and this added distance to the air-core increases coupling loss. Additionally, with a tapered fiber setup coupling into higher-order modes may be occurring and would increase loss.

### Temperature dependence

**Fig. 5:**
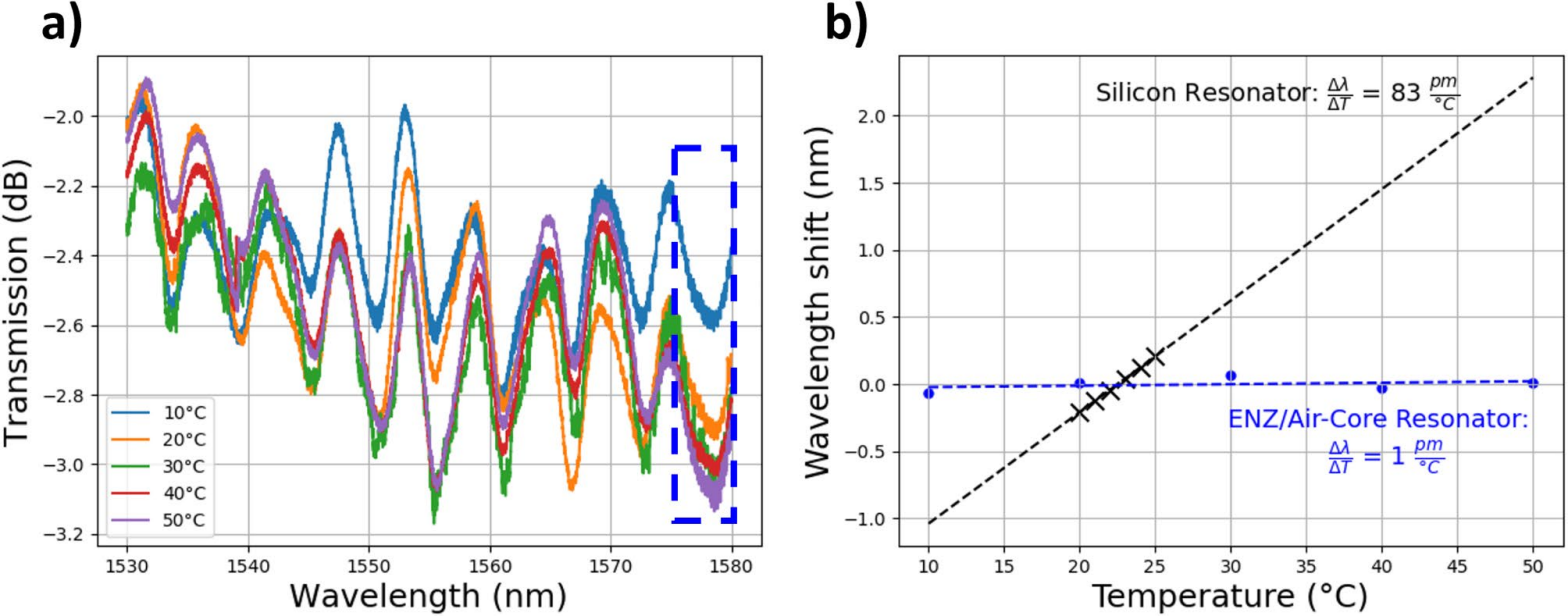
Demonstration of the athermal property of the resonators. (**a**), The transmission spectra at different temperatures. (**b**), The resonant dip location at each temperature for the resonance at 1578 nm (boxed in blue in **a**). A linear fit of these points shows a TDWS of ~ 1 pm/°C (blue line). Also shown is the TDWS of a Si resonator for comparison (black line).

Figure 5a shows the transmission spectra measured in the temperature range (10–50)°C. The spectra shown are the average of three spectra measured at each temperature. Changing the temperature affected the delicate coupling conditions and readjustment of the resonator position was needed to reestablish coupling during temperature sweeps. This led to the inconsistent trend in spectra power when measured at different temperatures. In addition to lowering the total loss, improvements to the coupling mechanism would reduce the variability in this measurement. The wavelength location of each of the resonances was found by curve fitting the data around the approximate location of each minima to a Lorentzian function. The center values of these fittings were used as the resonance wavelength locations.

Figure 5b shows the wavelength shift of the resonance at 1578 nm (outlined in blue) plotted as a function of temperature. The linear fit shows that the temperature dependent wavelength shift (TDWS) is ~ 1 pm/°C. The TDWS was measured for each of the resonances shown in Fig. 5a and they were found to vary from 1 to 15 pm/°C within the temperature range 10–50 °C. This variation is likely due to two things. First, the tapered fiber positioning is perturbed during the temperature sweeps which will affect the measurements and ultimately add uncertainty to the peak positions. Second, mode confinement decreases at wavelengths further from the ε_1_ = 0 point. This means more of the light travels through the ITO cladding which does not have very low TOC and the TDWS will have a slightly increased temperature dependence.

We compared the temperature sensitivity of the hollow-core resonator to a representative silicon-photonics ring resonator which was fabricated using the AIM Photonics Multi-Project Wafer (MPW) process. This silicon ring resonator has a waveguide width of 480 nm, height of 220 nm, coupling gap of 100 nm, and a ring radius of 5.24 µm. In this wavelength range the hollow-core resonator’s measured TDWS is around 50–80 times lower than the Si ring resonator’s TDWS of 83 pm/°C, as seen in (Fig. 5b). The TDWS of the hollow-core resonator is comparable to other athermal resonator designs which have shown TDWS of 0.14 pm/°C^10^, 2.1 pm/°C^13^, and 5.5 pm/°C^14^.

## Discussion

The discrepancy between our measured loaded Q (700) and the estimated high intrinsic Q (> 6000) indicates two things. First, we likely have high coupling loss caused by the ITO cladding material thickness and roughness that induces scattering. Second, the ε_1_ = 0 point of the ITO cladding on the measured device is probably not at the wavelengths where we conducted the measurements. Improvements to this device could be made by optimizing the coupling mechanism and the cladding thickness. A thick cladding is needed for structural stability and improved light confinement, but this also makes it difficult to couple light into the core which increases the coupling loss. Optimizing the annealing procedure would also be beneficial and allow the effective index to be tuned so that the maximum intrinsic Q is achieved in the desired operating wavelengths. Other cross-sectional geometries may also increase the Q. Therefore, there is a lot of room for improvements to be made to future devices.

In conclusion, we have demonstrated an ENZ-clad hollow-core resonator. The hollow-core gives this resonator reduced thermal sensitivity due to the much lower TOC of air compared to Si or other typical photonic materials. The ENZ cladding was made from ITO that was processed after deposition to drive its complex permittivity toward zero. The athermal property of the resonances persisted from 1530 to 1580 nm and the TDWS was 1 to 15 pm/°C depending on the wavelength. The tunability of the complex permittivity in ITO would allow a hollow-core microresonator to operate in other wavelength regimes if desired. Additionally, our method of 3D printing the resonator core has the added benefit of providing increased degrees of freedom when designing the shape of the core. Controlling the geometry of the core region could allow for dispersion control in the resonator^33^.

This approach to constructing an athermal optical resonator is intrinsically less sensitive to thermal fluctuations due to the hollow-core design. Additionally, an air or vacuum core will have essentially no stresses and strains that would normally affect the refractive index of a core made from layered solid materials. This potentially would make the device less sensitive to other environmental perturbations such as vibration if the device can be embedded into a rigid material like SiO_2_. The device shown in this work proves the concept and further optimizations could increase the Q by an order of magnitude and allow it to lock a high-Q oscillator in a reasonable amount of time.

## Methods

### Microring resonator fabrication

**Fig. 6:**
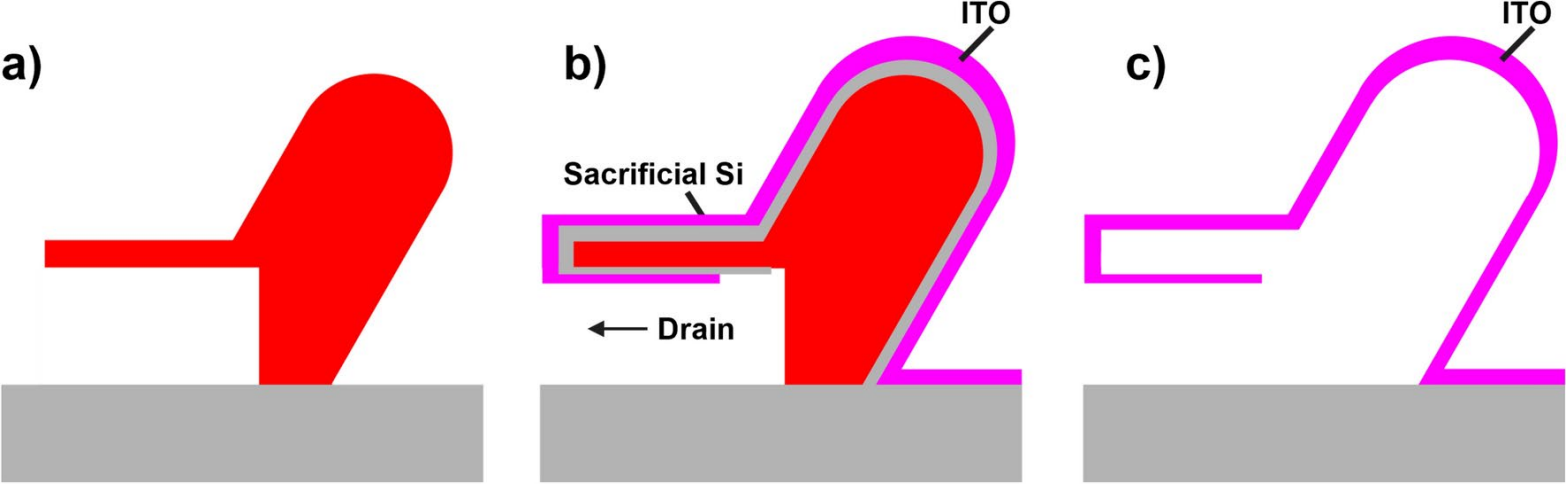
Steps to fabricate the hollow-core resonators. (**a**), The IP-Dip core is printed from a 3D CAD model using the Nanoscribe Photonic Professional GT2. (**b**), A sacrificial Si layer is deposited onto the core. The substrate was mounted onto a rotating stage at an angle steep enough to ensure even coverage around the MRR and to make sure adequate coverage inside the drain channels. The ITO layer was then deposited in a similar way, but at a shallow angle to make sure the sacrificial Si layer was still exposed in the drain. (**c**), The Si layer was removed with a XeF2 etch and then the IP-Dip core was ashed and removed via an annealing step. This final anneal step simultaneously processed the ITO to achieve an ENZ condition.

Fabrication of the MRR utilized a high resolution two-photon polymerization 3D printer from Nanoscribe (Photonic Professional GT2). This allows us to engineer a complex-shaped object out of photoresin (Nanoscribe IP-Dip) that formed the shape of the core region of the resonator which Si and ITO were later deposited onto. A 200 nm thick layer of sacrificial silicon was used to decouple the ITO from the IP-Dip core. When annealed, the IP-Dip shrinks before it ashes away and this shrinking would destroy the ITO layer unless decoupled with a sacrificial layer. Included also in the design were “drains” to act as channels to etch away the Si sacrificial layer and then remove the photoresin core, leaving behind an ITO clad hollow-core resonator. This process is illustrated in (Fig. 6).

The Si sacrificial layer was 200 nm thick and deposited using an Evatec BAK 641 E-beam evaporator along with a rotating sample mount to ensure uniform deposition around the MRR. This sacrificial layer was later removed using XeF_2_ etching. The ITO layer deposited onto the substrate was grown to 1600 nm and deposition was done via pulsed DC magnetron sputtering using a 90/10 In_2_O_3_/SnO_2_ wt % ITO target from Kurt J. Lesker (KJL). The ITO growth procedure is detailed more in Ref. 16. The thickness is expected to vary around the structure due to the differing angles of deposition. Measurements on test structures showed that the thickness varied from ~ 1.250 µm on the resonator sides to around ~ 3.3 µm at the top surface (Fig. 1b). Removing the photoresin core and processing the ITO films so they would have the appropriate optical properties were combined into one single annealing step. Post deposition annealing was done in an Allwin21 rapid thermal annealing tool. The annealing steps were done in a nitrogen environment with a N_2_ flow rate of 5 L/min at 650 °C. Once this was done, the sample was diced with a LPKF Protolaser U4 laser cutting tool in order to cut the substrate into sufficiently thin slices for measurement. This was needed so that enough clearance was present to allow the tapered fiber to come close enough to the microring resonators.

### Spectroscopic ellipsometry

We used an J.A. Woolam™ M-2000 variable angle ellipsometer and CompleteEASE (v. 6.51) analysis software. To model the ITO ellipsometry data, we used the Drude and Tauc-Lorentz (TL) model to find the real and imaginary permittivity, ε_1_ and ε_2_^34,35^. All SEM images were obtained with a ZEISS™ Auriga SEM.

### Testing setup

We used an Agilent 8164A Lightwave Measurement System with an 81682A tunable laser and an 81633A Power sensor. The output power from the laser was 5 dBm and was swept over the wavelength range of 1530–1580 nm. The sample was placed into an isolation box with a continuous flow of nitrogen to prevent dust from settling on the tapered fiber and to minimize condensation on the sample during low temperature testing. The tapered fiber was made using a Thorlabs Vytran Automated Glass Processor (GPX-3800). For testing, the sample was aligned to the tapered fiber and then brought closer for coupling. The power measured by the power meter was ~ -4.5 dBm for just the baseline tapered fiber measurements, indicating a ~ 9 dB loss in the system. This is mostly due to scattering of higher order modes in the tapered fiber.

## Supplementary Information

Supplementary Information.

## Data Availability

Data underlying the results presented in this paper are not publicly available at this time but may be obtained from the corresponding authors upon reasonable request.
