# Supplementary material for: Thermal insensitivity of an ENZ-ITO clad, hollow-core micro-ring resonator
Source: Sci Rep. 2025 Mar 29;15:10927. doi: 10.1038/s41598-024-82147-7 (PMC11954997; doi:10.1038/s41598-024-82147-7)
Supplement: Supplementary file 1 — Supplementary Information. [file 41598_2024_82147_MOESM1_ESM.docx]

**Supplementary Material**

**Thermal Insensitivity of an ENZ-ITO Clad, Hollow-core Micro-ring Resonator**

Andrew S. DeLoach*, Stephen R. Anderson, Sang-Yeon Cho, Jimmy H. Ni, Weimin Zhou*

DEVCOM Army Research Laboratory 2800 Powder Mill Rd, Adelphi MD, 20783

*email: andrew.s.deloach.civ@army.mil; weimin.zhou.civ@army.mil

S1. Table comparing the temperature dependent wavelength shift (TDWS) and other properties of the athermal resonators from this work and references 1,2, and 3 respectively.

| **Material** | **Radius (µm)** | **Wavelength (nm)** | **Q** | **TDWS (pm/°C)** | **Range (°C)** |
| --- | --- | --- | --- | --- | --- |
| Hollow-core [This work] | 65 | 1578 | 700 | 1 | 10-50 |
| TiO_2_/Si_3_N_4_^1^ | 200 | 1549.8 | 1.55 ∙ 10^5^ | 0.14 | 25-60 |
| Si/Polymer^2^ | 60 | 1551 | 1.25 ∙ 10^4^ | 2.1 | 25-55 |
| LN/TiO_2_^3^ | 100 | 1542 | 4.6 ∙ 10^5^ | 5.5 | -10-50 |

[1] Qiu, F., Spring, A. M. & Yokoyama, S. Athermal and High-Q Hybrid TiO2–Si3N4 Ring Resonator via an Etching-Free Fabrication Technique. ACS Photonics 2, 405-409 (2015).

[2] Qiu, F. et al. Athermal Hybrid Silicon/Polymer Ring Resonator Electro-optic Modulator. ACS Photonics 3, 780-783 (2016).

[3] Ling, J. et al. Athermal lithium niobate microresonator. Opt Express 28, 21682-21691 (2020).
